# Reduced environmental impact in body CT imaging with deep learning reconstruction: experience of a high-volume tertiary referral center

**DOI:** 10.1186/s13244-026-02333-1

**Published:** 2026-06-24

**Authors:** Paolo Niccolò Franco, Cesare Maino, Davide Gandola, Cammillo Talei Franzesi, Rocco Corso, Elena De Ponti, Davide Ippolito

**Affiliations:** 1 Department of Diagnostic Radiology, Fondazione IRCCS San Gerardo dei Tintori, Monza, Italy; 2 Department of Medical Physics, Fondazione IRCCS San Gerardo dei Tintori, Monza, Italy; 3 School of Medicine, University of Milano Bicocca, Monza, Italy

**Keywords:** Deep learning, Computed tomography, Sustainability, Iodinated contrast media, Low-kV imaging

## Abstract

**Purpose:**

To evaluate the environmental impact associated with CT scanners equipped with deep-learning-based image reconstruction (DLIR) compared with scanners equipped with hybrid-iterative reconstruction (HIR), focusing on electricity consumption, carbon dioxide equivalent (CO₂e) emissions, and iodinated contrast media (ICM) utilization in a high-volume tertiary referral center.

**Materials and methods:**

In this retrospective single-center study, environmental data were collected over an 18-month period from four CT scanners: two using HIR (Group 1) and two using DLIR (Group 2), including body CT examinations. DLIR-based protocols were implemented with reduced tube voltage (80–100 kV vs 120 kV) and optimized ICM doses. Electricity consumption, CO₂e emissions, and ICM utilization were quantified and compared between groups. Environmental outcomes were analyzed at the scanner level and normalized per examination.

**Results:**

A total of 42,300 examinations were analyzed (23,096 in Group 1; 19,204 in Group 2). Electricity consumption was 123,000 kWh for Group 1 and 66,927 kWh for Group 2, corresponding to 30.75 and 16.73 tons of CO₂e emissions, respectively. At the scanner level, this represented a reduction of 28,037 kWh and 7.01 tons of CO₂e per scanner (4.67 tons/year). DLIR-based protocols were associated with an ICM saving of 434 L over 18 months, corresponding to 4.47 tons of avoided CO₂e emissions and 60,730 L of water preserved. Combined CO₂e emissions from electricity and ICM were 49.62 tons in Group 1 and 29.10 tons in Group 2.

**Conclusion:**

DLIR-based optimized protocols were associated with improved environmental metrics, supporting their potential contribution to more sustainable radiology practices in high-volume settings.

**Clinical relevance statement:**

Deep learning-based image reconstruction enables routine body CT protocols with lower tube voltage and reduced ICM dose, supporting a clinically feasible transition toward more sustainable CT practice in high-volume imaging workflows.

**Key Points:**

- DLIR was associated with the implementation of lower tube voltage and reduced ICM dose, supporting more sustainable CT imaging based on protocol adaptations.
- In a high-volume tertiary referral center, deep learning-based image reconstruction was associated with a substantial reduction in electricity consumption and overall CO₂-equivalent emissions compared with hybrid iterative reconstruction.
- Optimization of contrast media dosing with deep learning-based image reconstruction contributed meaningfully to environmental benefits.

**Graphical Abstract:**

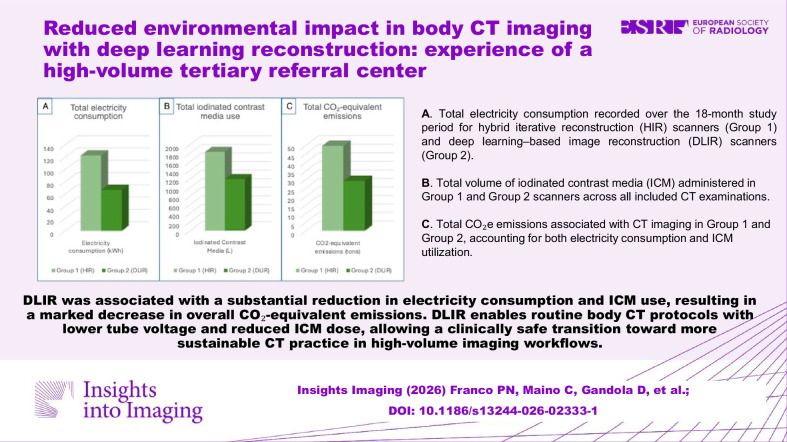

## Introduction

In recent years, growing attention has been directed toward the environmental footprint of healthcare, with a particular focus on medical imaging, which is a main contributor to hospital energy consumption and related carbon dioxide equivalent (CO₂e) emissions [1, 2]. In particular, CT plays a key role due to its high electrical demand, extensive worldwide use, and reliance on iodinated contrast media (ICM) [3, 4]. As a result, imaging departments are increasingly challenged to limit resource consumption without affecting diagnostic accuracy [5].

Over time, technical developments in CT reconstruction have primarily focused on reducing radiation dose while preserving image quality. Hybrid-iterative reconstruction (HIR) and model-based iterative reconstruction (MBIR) have enabled substantial dose savings [6, 7]. However, these techniques are demanding on computing resources and may affect scanner energy usage as well as workflow efficiency, particularly reconstruction time [8]. More recently, deep learning-based image reconstruction (DLIR) has been introduced as an alternative approach, providing low-noise images with improved image quality and reducing the reconstruction time [9, 10]. An additional advantage of DLIR lies in its ability to support low-tube voltage imaging, which is particularly important from a sustainability perspective, since CT energy consumption rises quadratically with tube voltage [11–13]. Moreover, lower voltage CT imaging increases iodine attenuation, allowing an intrinsic possible reduction in ICM dose without loss of image definition [14–16].

Beyond electricity consumption, the production and disposal of ICM represent a considerable and often underestimated environmental burden. Iodine extraction, pharmaceutical manufacturing, and wastewater treatment contribute substantially to the overall carbon footprint of CT imaging [17–19]. Consequently, strategies that combine reduced tube voltage with lower ICM doses may offer further important sustainability benefits [4, 20].

Despite these potential advantages, real-world data on the environmental impact of DLIR implementation in high-volume clinical settings remain underreported. In particular, comprehensive evaluations addressing electricity consumption, CO₂e emissions, and ICM savings are still limited.

On these bases, the present study aims to assess the environmental impact of DLIR compared with HIR in a tertiary referral center. We focus on scanner electricity consumption, associated CO₂e emissions, and ICM savings, with the goal of clarifying whether DLIR can support more environmentally sustainable CT practice.

## Materials and methods

### Study design and setting

This retrospective, single-center observational study was conducted in a tertiary referral hospital equipped with four multidetector CT scanners. Two scanners belonged to an older generation (Group 1) and consisted of 256-slice systems (iCT, Philips Healthcare) equipped with a hybrid iterative reconstruction (HIR) algorithm (iDose, Philips Healthcare). The other two scanners (Group 2) were recently installed and were 128-slice systems (CT 5300, Philips Healthcare, Best, The NL) incorporating a DLIR system (PRECISE Image, Philips Healthcare).

**Fig. 1:**
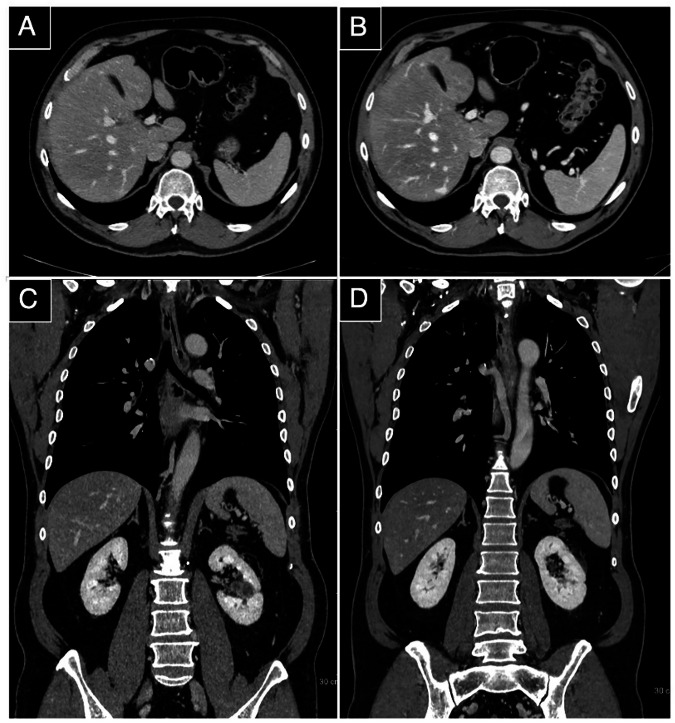
Contrast-enhanced CT axial images reconstructed with HIR (**A**) and DLIR (**B**), and coronal images reconstructed with HIR (**C**) and DLIR (**D**) are shown in the same patient. HIR acquisitions were performed at 120 kV with an ICM dose of 1.5 mL/kg, whereas DLIR acquisitions were performed at 100 kV with a reduced ICM dose of 1.2 mL/kg. Despite the lower tube voltage and reduced ICM dose, DLIR images appear comparable with HIR ones. HIR, hybrid iterative reconstruction; DLIR, deep learning-based image reconstruction; ICM, iodinated contrast media

**Fig. 2:**
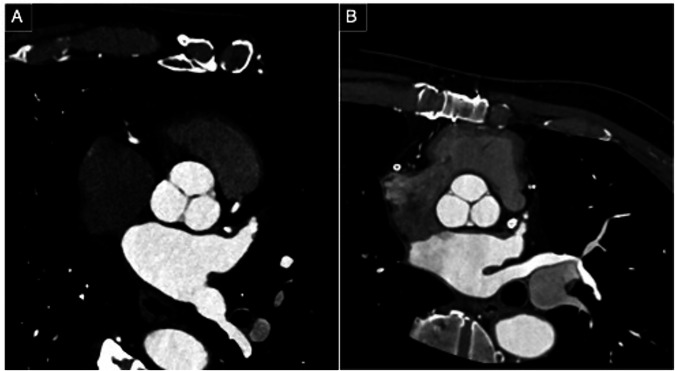
Oblique CT angiographic images of aortic sinuses acquired with a scanner equipped with HIR after administration of 70 mL of ICM (**A**), and with DLIR (**B**) after administration of 50 mL of ICM. CTA, computed tomography angiography; HIR, hybrid iterative reconstruction; ICM, iodinated contrast media; DLIR, deep learning-based image reconstruction

All CT body examinations (including neck, thorax, abdomen, and pelvis) of patients with a body mass index (BMI) < 30 performed on any of the four scanners over an 18-month period (January 2024 – June 2025) were included, irrespective of clinical indication or anatomical region (Figs. 1, 2). Patients with a BMI ≥ 30 were excluded to limit variability in acquisition parameters and reduce potential confounding related to energy consumption and contrast media utilization. Neuroimaging examinations—namely non-contrast and contrast-enhanced brain CT, CT angiography of the supra-aortic vessels, and spine CT—were excluded from the analysis, as neuroradiology examinations are mostly performed on dedicated scanners within a separate neuroradiology unit at our Institution. CT examinations of skeletal segments and extremities were also excluded from the analysis.

### CT examinations

Standardized acquisition protocols were applied to Group 1 scanners according to routine clinical practice. Included CT examination protocols were: CT pulmonary angiography, aortic CT angiography, coronary CT angiography, contrast-enhanced abdomino–pelvic CT, high-resolution chest CT, oncologic whole-body CT (including neck, chest, abdomen, and pelvis contrast-enhanced single-phase scan), trauma CT (consisting of an un-enhanced scan including chest, abdomen, and pelvis, and a contrast-enhanced multiphase examination, with arterial and venous phases and a delayed acquisition when clinically indicated), and pediatric CT protocols (including neck, chest, abdomen, and pelvis contrast-enhanced). Most examinations were performed using a tube voltage of 120 kV, with tube current modulation adapted to patient size and clinical indication.

ICM was administered intravenously by using a dosage of 1.5 mL/kg for contrast-enhanced abdomino–pelvic CT, oncologic whole-body CT, and trauma CT. For CT pulmonary angiography, aortic CT angiography, and coronary CT angiography, a fixed dose of ICM was administered, particularly 60 mL, 60 mL and 80 mL. For pediatric protocols, the dosage was 1 mL/kg.

On Group 2 scanners, DLIR-based protocols were standardized and derived from the corresponding HIR protocols by selectively optimizing acquisition parameters while keeping scanner-dependent settings unchanged. Tube voltage was systematically reduced to 80 or 100 kV depending on examination type. Based on the improved contrast-to-noise ratio achievable with DLIR at lower tube voltages, contrast-enhanced protocols were moderately adjusted. Namely, ICM was administered intravenously by using a dosage of 1.2 mL/kg for contrast-enhanced abdomino–pelvic CT, oncologic whole-body CT, and trauma CT. For CT pulmonary angiography, aortic CT angiography, and coronary CT angiography, a fixed dose of ICM was administered, particularly 40 mL, 40 mL and 60 mL, respectively. For pediatric protocols, the dosage was 0.8 mL/kg.

For both protocols, the same contrast media was used (Iobiditrol 350—Guerbet). Injection flow rate and timing were preserved. All acquisition and reconstruction parameters were predefined and standardized across examinations.

Group 1 and Group 2 detailed protocol-specific parameters for each clinical indication are reported in Table 1.

**Table 1:** CT acquisition and reconstruction parameters for the evaluated clinical protocols reconstructed with HIR and DLIR

CT protocol	HIR (Group 1)	DLIR (Group 2)
Pulmonary embolism CTA		
Tube-voltage (kV)	100	80
Tube-current (mAs)	Automated	Automated
Gantry rotation time (s)	0.33	0.27
Collimation	128 × 0.625	128 × 0.625
Pitch	0.765	0.765
Thickness/increment (mm)	1/0.5	1/0.5
Flow rate (mL/s)	3.5	3.5
Aortic CT (chest/abdomen/pelvis)		
Tube-voltage (kV)	100	80
Tube-current (mAs)	Automated	Automated
Gantry rotation time (s)	0.75	0.5
Collimation	128 × 0.625	128 × 0.625
Pitch	0.30	0.30
Thickness/increment (mm)	0.8/0.4	0.6/0.3
Flow rate (mL/s)	3.5	3.5
Cardiac CT/coronary CTA		
Tube-voltage (kV)	100	80
Tube-current (mAs)	Automated	Automated
Gantry rotation time (s)	0.33	0.27
Collimation	128 × 0.625	128 × 0.625
Pitch	0.16–0.24 (ECG-gated)	0.16–0.24 (ECG-gated)
Thickness/increment (mm)	0.8/0.4	0.6/0.3
Flow rate (mL/s)	4.5	4.5
Abdomino-pelvic CT		
Tube-voltage (kV)	120	100
Tube-current (mAs)	Automated	Automated
Gantry rotation time (s)	0.75	0.5
Collimation	128 × 0.625	128 × 0.625
Pitch	0.98	0.98
Thickness/increment (mm)	2.0 /1.0	2.0/1.0
Flow rate (mL/s)	3.5	3.5
High-resolution chest CT		
Tube-voltage (kV)	120	100
Tube-current (mAs)	Automated	Automated
Gantry rotation time (s)	0.5	0.33
Collimation	128 × 0.625	128 × 0.625
Pitch	1.2	1.2
Thickness/increment (mm)	1.0/0.5	0.6/0.5
Oncologic whole-body CT		
Tube-voltage (kV)	120	100
Tube-current (mAs)	Automated	Automated
Gantry rotation time (s)	0.75	0.5
Collimation	128 × 0.625	128 × 0.625
Pitch	0.91	0.91
Thickness/increment (mm)	2.0/1.0	2.0/1.0
Flow rate (mL/s)	3.5	3.5
Trauma whole-body CT		
Tube-voltage (kV)	120	100
Tube-current (mAs^*^)	Automated	Automated
Gantry rotation time (s)	0.75	0.5
Collimation	128 × 0.625	128 × 0.625
Pitch	0.91	0.91
Thickness/increment (mm)	2.0/1.0	2.0/1.0
Flow rate (mL/s)	3.5	3.5
Pediatric whole-body CT		
Tube-voltage (kV)	100	80
Tube-current (mAs^*^)	Automated	Automated
Gantry rotation time (s)	0.75	0.5
Collimation	128 × 0.625	128 × 0.625
Pitch	1.2	1.2
Thickness/increment (mm)	1.0/0.5	1.0/0.5
Flow rate (mL/s)	3.5	3.5

*HIR* hybrid iterative reconstruction, *DLIR* deep learning-based image reconstruction, *ICM* iodinated contrast media, *CTA* computed tomography angiography, *ECG* electrocardiogram

### Data collection and environmental impact assessment

All CT examinations acquired during the study period were retrieved from the radiological information system and picture archiving and communication system (PACS—Agfa Enterprise Imaging, Agfa HealthCare) database, including scanner identification, examination category, acquisition parameters, and total ICM volume administered.

Energy consumption for each scanner was obtained from the hospital engineering department. Continuous power monitoring systems provided annual kWh values for each device. Scanner-level electricity consumption was directly measured, whereas protocol-level attribution was used only for normalization purposes. For protocols using multiple tube potentials, energy use was estimated according to the known quadratic relationship between tube voltage and power demand, corroborated with manufacturer specifications.

Annual CO₂e emissions associated with electricity consumption were calculated using the regional conversion factor applied by the hospital’s sustainability office, derived from official data published by the Italian Institute for Environmental Protection and Research (ISPRA) for the Institute region [21].

ICM-related environmental impact was assessed by summing the yearly contrast volume administered on each scanner and converting this value into CO₂e based on published life-cycle analyses of the pharmaceutical production and disposal chain.

Environmental benefits associated with DLIR adoption were quantified by comparing Group 2 with Group 1 in terms of: electricity savings (kWh/year), CO₂e reduction linked to lower energy use, annual ICM savings (litres/year), and associated reduction in ICM-related CO₂e.

Descriptive statistics were used to summarize examination volume, energy consumption, CO₂e emissions, and ICM utilization for both groups. Differences between groups were reported as absolute and relative changes.

The relationship between tube voltage and energy demand was assessed using the well-established principle that X-ray tube output increases in a non-linear, near-quadratic manner as the kilovoltage rises:

$${P\propto {kV}}^{2}$$

This behavior, widely described in CT physics literature [13, 22, 23], was used to estimate the relative reduction in electrical power associated with low-kV DLIR protocols compared with the 120-kV standard. These estimates were used for comparative and normalization purposes only and do not represent direct measurements of protocol-specific energy consumption.

CO₂e emissions related to electricity consumption were calculated by applying the regional location-based emission factor (EF), in accordance with the GHG Protocol Scope 2 Guidance, the standard framework for greenhouse gas accounting developed by the World Resources Institute (WRI) and the World Business Council for Sustainable Development (WBCSD) [24]:

$${{CO}}_{2}{{e}}\left({tons}\right)={kWh}\times {EF}$$

The location-based EF was calculated from the national electricity grid mix for the year 2023, based on publicly available national energy statistics reported by the International Energy Agency (IEA) [25].

ICM savings were calculated by comparing protocol-specific contrast media volumes between standard HIR-based acquisitions and optimized DLIR low-kV protocols. For each contrast-enhanced examination, the difference in ICM volume per scan was derived from the standardized protocol parameters, and total savings were obtained by multiplying this difference by the number of examinations performed for each protocol during the study period. To remove the effect of protocol-level ICM optimization from differences in examination distribution among the two Groups, a reference-equivalent analysis was performed by applying both HIR- and DLIR-specific ICM doses to the same Group 2 examination case-mix.

The environmental impact associated with the avoided production and utilization of ICM was quantified using published life-cycle supply-chain data, which takes into account iodine extraction, pharmaceutical manufacturing, distribution, and waste management. CO₂e emissions were estimated by applying an EF of 10.3 kg of CO₂e per liter of ICM [26]. For between-group comparisons of total CO₂e emissions, ICM–related CO₂e emissions were calculated for both Group 1 and Group 2 by applying this EF to the total administered contrast volume in each group.

Water savings were estimated using an assumed water-use factor of 140 L per liter of ICM, derived from life-cycle considerations of pharmaceutical manufacturing processes, which are known to be water-intensive due to purification and sterilization requirements [27]. As no ICM-specific water footprint data are currently available, uncertainty was explored through one-way sensitivity analyses using ±50% bounds (70–210 L/L), in accordance with ISO 14046 recommendations [28].

### Statistical analysis

Statistical analyses were performed according to the type of data analyzed. The distribution of CT examination categories between Group 1 and Group 2 was compared using inferential statistics. Categorical variables were evaluated through the chi-square (χ²) test, and odds ratios (ORs) with 95% confidence intervals (CIs) were calculated for each CT examination category using 2 × 2 contingency tables. A two-sided *p*-value < 0.05 was considered statistically significant.

Environmental outcomes, including electricity consumption, CO₂e emissions, and ICM utilization, were evaluated descriptively at the scanner-group level. As these metrics were derived from aggregated scanner-level data rather than from independent patient-level observations, no inferential statistical tests were applied. Results are therefore presented as absolute values and normalized per scanner, per examination, and per year where appropriate.

## Results

A total of 42,300 examinations were performed across the four CT scanners. Of these, 23,096 examinations (54.6%) were acquired on Group 1 scanners using HIR reconstruction, whereas 19,204 examinations (45.4%) were performed on Group 2 scanners equipped with DLIR. The distribution of CT examination categories differed between the two groups (*p* < 0.001). The study population had a mean age of 64 ± 15 years (range: 2–93 years) in Group 1 and 58 ± 17 (range: 1–91 years) in Group 2, including both adult and pediatric patients. Detailed distribution of CT examinations by protocol in Group 1 and Group 2 during the study period is reported in Table 2.

**Table 2:** Distribution of CT examinations by protocol in Group 1 and Group 2 over the 18-month study period

CT examination	Group 1 (*n* = 23,096)	Group 2 (*n* = 19,204)	*p*-value
Pulmonary embolism CTA	723 (3.1%)	1392 (7.2%)	< 0.001
Aortic CTA (chest/abdomen/pelvis)	494 (2.1%)	1178 (6.1%)	< 0.001
Cardiac CT/coronary CTA	317 (1.4%)	1050 (5.5%)	< 0.001
Abdomino–pelvic CT	6409 (27.7%)	7659 (39.9%)	< 0.001
High-resolution chest CT	4497 (19.5%)	2745 (14.3%)	< 0.001
Oncologic whole-body CT	9298 (40.3%)	2550 (13.3%)	< 0.001
Trauma whole-body CT	637 (2.8%)	1674 (8.7%)	< 0.001
Pediatric CT	721 (3.1%)	956 (5.0%)	< 0.001

Values represent the actual number of examinations performed in each category

*CTA* computed tomography angiography

### Energy consumption and CO₂e emissions

**Fig. 3:**
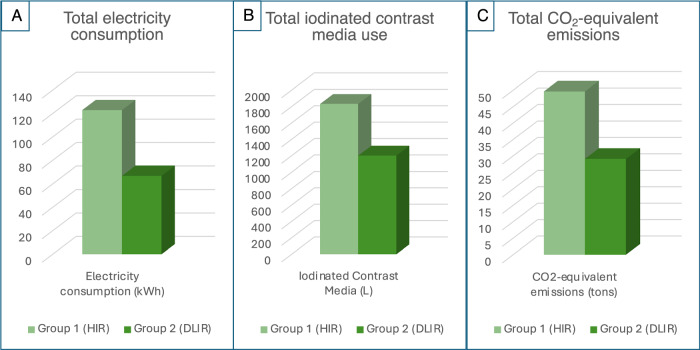
**A** Total electricity consumption was recorded over the 18-month study period for Group 1 and Group 2 scanners. **B**. Total volume of ICM administered in Group 1 and Group 2 scanners across all included CT examinations. **C**. Total CO₂e emissions associated with CT imaging in Group 1 and Group 2, accounting for both electricity consumption and ICM utilization. HIR, hybrid iterative reconstruction; DLIR, deep learning-based image reconstruction; ICM, iodinated contrast media

Over the 18-month study period, Group 1 exhibited a total electricity consumption of 123,000 kWh, corresponding to 30.75 tons of CO₂e emissions based on the regional electricity EF. In contrast, Group 2 showed a significantly lower total electricity consumption of 66,927 kWh, resulting in 16.73 tons of CO₂e emissions (*p* < 0.001) (Fig. 3A). This represented an absolute reduction of 56,073 kWh and 14.02 tons of CO₂e in favor of DLIR-equipped scanners.

When expressed at the scanner level, mean electricity consumption was 61,500 kWh per scanner for Group 1 and 33,463 kWh per scanner for Group 2 (*p* < 0.001), corresponding to 15.38 and 8.37 tons of CO₂e per scanner, respectively (*p* < 0.001). Overall, Group 2 achieved a net reduction of 28,037 kWh and 7.01 tons of CO₂e per scanner over the study period, corresponding to annual reductions of 18,691 kWh and 4.67 tons of CO₂e per scanner.

To account for differences in scanner workload between the two groups, electricity consumption was further normalized per CT examination. Group 1 scanners consumed 5.33 kWh per examination, whereas Group 2 scanners required 3.49 kWh per examination (*p* < 0.001), corresponding to a 34.5% reduction in energy demand per scan. In terms of carbon emissions, this translated into 1.33 kg of CO₂e per examination for Group 1 compared with 0.87 kg of CO₂e per examination for Group 2 (*p* < 0.001).

### Contrast media utilization

The total amount of ICM administered over the study period was 1832 L for Group 1 scanners and 1201 L for Group 2 scanners (Fig. 3B), corresponding to 18.87 and 12.37 tons of CO₂e, respectively. The observed difference in total administered ICM between the two groups reflects both variations in examination case-mix and differences in protocol-specific contrast dosing. To isolate the effect of protocol-level contrast dose optimization obtained by DLIR independently of examination distribution, a reference-equivalent analysis was therefore performed by applying both HIR- and DLIR-specific ICM to the same Group 2 case-mix. Based on this approach, the lower nominal contrast doses adopted on DLIR scanners resulted in an exact reference-equivalent contrast saving of 434 L, corresponding to 289 L per year. When translated into environmental impact metrics, this ICM reduction corresponded to 4.47 tons of CO₂e emissions avoided over the 18-month period, equal to 2.98 tons of CO₂e per year.

In addition, the reduced ICM utilization was associated with 60,730 L of water preserved over 18 months, corresponding to 40,486 L per year.

### Combined environmental benefit

When electricity consumption and ICM use were considered together, Group 1 scanners were associated with higher total CO₂e emissions over the 18-month study period compared with Group 2. Overall, the combined CO₂e emissions from electricity consumption and ICM utilization totaled 49.62 tons for Group 1 and 29.10 tons for Group 2 over the 18-month study period (*p* < 0.001) (Fig. 3C). When normalized per year, this corresponded to 33.08 tons of CO₂e per year for Group 1 and 19.40 tons of CO₂e per year for Group 2 (*p* < 0.0001). These findings indicate a substantially lower overall environmental impact associated with DLIR-equipped scanners in routine clinical practice.

Comparison of energy consumption, CO₂e emissions, and ICM doses between Group 1 and Group 2 are summarized in Table 3.

**Table 3:** Comparison of energy consumption, CO₂e emissions, and ICM utilization between Group 1 (HIR) and Group 2 (DLIR) scanners

Parameter	Group 1 (HIR)	Group 2 (DLIR)	*p*-value
Total electricity consumption (kWh)	123,000	66,927	< 0.001
Electricity consumption per scanner (kWh)	61,500	33,463	< 0.001
Electricity consumption per examination (kWh)	5.33	3.49	< 0.001
CO₂e emissions from electricity (tons)	30.75	16.73	< 0.001
CO₂e emissions per scanner (tons)	15.38	8.37	< 0.001
CO₂e emissions per examination (kg)	1.33	0.87	< 0.001
Total ICM volume administered (l)	1832	1201	< 0.001

*HIR* hybrid iterative reconstruction, *DLIR* deep learning-based image reconstruction, *ICM* iodinated contrast media, *CO₂e* carbon dioxide–equivalent

Reference-equivalent ICM saving was calculated by applying Group 1 and Group 2 protocol-specific contrast media doses to the Group 2 examination case-mix, in order to isolate the effect of protocol-level contrast dose optimization independently of differences in examination distribution

## Discussion

In this real-world study performed in a high-volume tertiary referral center, the introduction of DLIR was associated with a significant decrease in the environmental impact of routine CT imaging compared with HIR ones. The DLIR-based protocols evaluated in this study reflect routine clinical practice in our Institution, supporting the real-world applicability of the findings.

Importantly, the observed environmental benefits should not be attributed solely to the reconstruction algorithm itself. In our study, DLIR should be interpreted as an enabling technology that facilitates the implementation of optimized acquisition protocols, including lower tube voltage and reduced ICM dosing. Therefore, the reported reductions in energy consumption and ICM utilization likely reflect the combined effect of technological advancements and protocol modifications, rather than the isolated impact of DLIR alone.

To date, the majority of the literature on DLIR has concentrated on image quality and radiation dose [29–32], with less attention paid to its potential environmental implications, such as energy consumption, carbon emissions, and ICM dose.

Due to the high-power needs of multidetector scanners and their extensive use in routine clinical practice, electricity consumption continues to be a significant factor in the environmental impact of CT. Our study’s observed reduction is in line with the widely accepted near-quadratic relationship between tube voltage and X-ray tube power output [13, 22]. This further supports the interpretation that protocol-level changes, particularly tube voltage reduction, play a major role in determining energy consumption, with DLIR acting as a facilitating factor. In fact, our findings suggest that the same technical features that enable low-kV imaging may also lead to lower energy demand in routine clinical use. Significantly, these reductions were observed in a wide spectrum of CT examinations, reflecting everyday departmental activity rather than highly selected or experimentally optimized protocols.

Although direct comparisons are still difficult due to variations in study designs and measurement approaches, some recently published papers provide some context for our findings. The majority of studies regarding CT-related energy usage are based on single-scanner measurements or experimental settings designed to isolate technical factors that affect power consumption. Schoen et al reported a correlation between acquisition parameters and net scan energy consumption using direct electrical measurements, supporting the idea that protocol-level optimization can result in quantifiable energy savings [33]. Other authors have examined strategies, such as rapid-reactivation power-saving modes during short periods, underlining that non-scan energy consumption may also contribute substantially to the overall footprint in busy departments, although the benefits are highly dependent on workflow characteristics [34]. Systematic reviews have demonstrated that CT is one of the main contributors to modality-related energy demand in radiology departments [2, 3]. However, real-world data connecting specific technical solutions to quantifiable carbon reductions is scarce [3]. Moreover, life-cycle assessment methods show that scanner electricity represents only one part of imaging-related emissions, highlighting the relevance of comprehensive assessments that consider multiple environmental factors [35].

In addition to electricity use, ICM represents an additional and often overlooked source of environmental burden in CT imaging [17, 20]. The production, distribution, and disposal of contrast agents are highly energy-intensive processes and contribute to both carbon emissions and water consumption [17, 19]. In the present study, DLIR protocols were associated with a reduction in administered contrast volume across multiple contrast-enhanced protocols due to the intrinsic improved contrast-to-noise ratio at lower tube voltages [36]. This was associated with a quantifiable decrease in contrast-related CO₂e emissions and water consumption. Even though the absolute carbon savings generated by contrast reduction were lower than those related to electricity consumption, they remain meaningful from a sustainability perspective and should be taken into consideration when evaluating the sustainability of imaging.

Previous studies have demonstrated the feasibility of ICM reduction in selected protocols, such as CT pulmonary angiography and oncologic imaging, especially when low-kV techniques are applied [7, 16]. These findings are further supported by our results, which highlight that DLIR allows contrast optimization strategies applicable at the departmental scale without modifying workflows. This may have practical relevance, as sustainability strategies that do not require changes in indications or workflow are more likely to be adopted and maintained over time [37].

The increasing attention on environmental sustainability in radiology has largely focused on energy efficiency, scanner technology, and radiation dose optimization [1, 2]. While these factors remain central, our findings emphasize the significance of taking consumables such as ICM into account when assessing the overall environmental impact of CT imaging. From a practical point of view, the reductions observed in this study were attained as a secondary benefit of technological advancements implemented for clinical purposes rather than through specific “green” protocols.

It should also be noted that the training of deep learning models requires substantial computational resources and energy consumption, which may contribute to the overall environmental footprint of artificial intelligence (AI) [38]. Although this aspect was not evaluated in the present study, it represents an important consideration when assessing the net sustainability of AI-based imaging technologies.

The present study has several limitations. Firstly, its retrospective and single-center design may limit generalizability, as scanner models, energy mixes, and clinical workflows vary across Institutions. The study design does not allow disentangling the individual contribution of DLIR from that of protocol modifications and scanner generation differences, limiting causal inference. Although scanner-level electricity consumption was directly measured, protocol-level energy attribution relied on modeled estimates and was used for normalization only. Per-examination energy values should therefore be interpreted as estimates based on established physical relationships, not direct measurements. Similarly, environmental impact estimates for ICM were derived from published life-cycle assessments and should be interpreted as estimates. Differences in scanner architecture and generation may have influenced acquisition parameters and energy consumption, representing a potential source of variability that cannot be fully separated from protocol-related effects. Although contrast media dosing was standardized using weight-based protocols, lean body weight (LBW)-based contrast administration was not applied, which could allow further individualization of contrast volume and potentially enhance both clinical and environmental benefits. Variability in examination case-mix between groups may represent a source of confounding, as variations in scan type and protocol can influence energy consumption and ICM utilization, thereby limiting causal inference. However, the use of reference-equivalent analysis in the present work minimizes the impact of patient size distribution, supporting that the observed ICM savings are primarily driven by DLIR-enabled protocol optimization rather than anthropometric differences. Finally, the high image quality associated with DLIR is inferred from existing literature, as this study did not directly evaluate or compare image quality between DLIR and prior reconstruction systems.

To conclude, the implementation of DLIR in routine CT practice was associated with a significant decrease in environmental impact, likely reflecting the combined effect of both lower electricity consumption and reduced ICM use. ICM dose optimization was a substantial contributor to carbon savings, although energy efficiency accounted for the majority. These findings support DLIR not only as a tool potentially improving image quality and dose efficiency, as suggested by prior literature, but also as a potential contributor to more environmentally sustainable CT imaging.

## Data Availability

The datasets generated and/or analyzed during the current study are not publicly available due to institutional and data protection regulations, but are available from the corresponding author on request.

## Abbreviations

CO₂e: Carbon dioxide equivalent
DLIR: Deep-learning-based image reconstruction
EF: Emission factor
HIR: Hybrid-iterative reconstruction
IEA: International Energy Agency
ICM: Iodinated contrast media
ISPRA: Italian Institute for Environmental Protection and Research
WBCSD: World Business Council for Sustainable Development
WRI: World Resources Institute

## References

[1] Picano E, Mangia C, D’Andrea A (2022) Climate change, carbon dioxide emissions, and medical imaging contribution. J Clin Med 12:215. 10.3390/jcm12010215 36615016 10.3390/jcm12010215 PMC9820937

[2] Thiel CL, Vigil-Garcia M, Nande S et al (2024) Environmental life cycle assessment of a U.S. hospital-based radiology practice. Radiology 313:e240398. 10.1148/radiol.240398 39589247 10.1148/radiol.240398 PMC11605107

[3] Roletto A, Zanardo M, Bonfitto GR et al (2024) The environmental impact of energy consumption and carbon emissions in radiology departments: a systematic review. Eur Radiol Exp 8:35. 10.1186/s41747-024-00424-6 38418763 10.1186/s41747-024-00424-6 PMC10902235

[4] Vosshenrich J, Merkle EM, Heye T (2025) The carbon footprint of modern imaging. Curr Opin Urol 35:674–678. 10.1097/MOU.0000000000001337 40905239 10.1097/MOU.0000000000001337 PMC12517713

[5] Toker I, Jansen S, Knoche J, Lorenz D (2025) Digital innovations for sustainable radiology: reducing waste, emissions, and inefficiencies in imaging workflows. Digit Health 11:20552076251386788. 10.1177/20552076251386788 41122428 10.1177/20552076251386788 PMC12536090

[6] Ippolito D, Maino C, Pecorelli A et al (2021) Application of low-dose CT combined with model-based iterative reconstruction algorithm in oncologic patients during follow-up: dose reduction and image quality. Br J Radiol 94:20201223. 10.1259/bjr.20201223 34233459 10.1259/bjr.20201223 PMC8764930

[7] Ippolito D, De Vito A, Franzesi CT et al (2019) Evaluation of image quality and radiation dose saving comparing knowledge model-based iterative reconstruction on 80-kV CT pulmonary angiography (CTPA) with hybrid iterative reconstruction on 100-kV CT. Emerg Radiol 26:145–153. 10.1007/s10140-018-1653-4 30415416 10.1007/s10140-018-1653-4

[8] Silva AC, Lawder HJ, Hara A et al (2010) Innovations in CT dose reduction strategy: application of the adaptive statistical iterative reconstruction algorithm. AJR Am J Roentgenol 194:191–199. 10.2214/AJR.09.2953 20028923 10.2214/AJR.09.2953

[9] Chen H, Li Q, Zhou L, Li F (2024) Deep learning-based algorithms for low-dose CT imaging: a review. Eur J Radiol 172:111355. 10.1016/j.ejrad.2024.111355 38325188 10.1016/j.ejrad.2024.111355

[10] Maino C, Franco PN, Szafranska E et al (2026) Improved image quality and dose reduction in liver CT using deep learning-based reconstruction: a comparative study. Eur J Radiol 194:112520. 10.1016/j.ejrad.2025.112520 41264979 10.1016/j.ejrad.2025.112520

[11] Yang C-C (2020) Evaluation of impact of factors affecting CT radiation dose for optimizing patient dose levels. Diagnostics (Basel) 10:787. 10.3390/diagnostics10100787 33028021 10.3390/diagnostics10100787 PMC7600150

[12] Chang KJ, Collins S, Li B, Mayo-Smith WW (2017) Optimizing CT technique to reduce radiation dose: effect of changes in kVp, iterative reconstruction, and noise index on dose and noise in a human cadaver. Radiol Phys Technol 10:180–188. 10.1007/s12194-016-0382-1 27699635 10.1007/s12194-016-0382-1

[13] McCollough CH, Primak AN, Braun N et al (2009) Strategies for reducing radiation dose in CT. Radiol Clin North Am 47:27–40. 10.1016/j.rcl.2008.10.006 19195532 10.1016/j.rcl.2008.10.006 PMC2743386

[14] Lin X, Gao Y, Zhu C et al (2024) Improved overall image quality in low-dose dual-energy computed tomography enterography using deep-learning image reconstruction. Abdom Radiol (NY) 49:2979–2987. 10.1007/s00261-024-04221-y 38480547 10.1007/s00261-024-04221-y

[15] Huang X, Shang J, Xiao Y et al (2025) Deep learning-based reconstruction improves image quality in low-dose head CT angiography. Malawi Med J 37:109–114. 10.4314/mmj.v37i2.8 41306806 10.4314/mmj.v37i2.8 PMC12538262

[16] Svalkvist A, Fagman E, Vikgren J et al (2023) Evaluation of deep-learning image reconstruction for chest CT examinations at two different dose levels. J Appl Clin Med Phys 24:e13871. 10.1002/acm2.13871 36583696 10.1002/acm2.13871 PMC10018655

[17] Toia GV, Ananthakrishnan L (2025) The environmental impact of iodinated contrast media: strategies for optimized use and recycling. J Comput Assist Tomogr 49:203–214. 10.1097/RCT.0000000000001674 39631428 10.1097/RCT.0000000000001674

[18] Yan H, Zhang T, Yang Y et al (2024) Occurrence of iodinated contrast media (ICM) in water environments and their control strategies with a particular focus on iodinated by-products formation: a comprehensive review. J Environ Manage 351:119931. 10.1016/j.jenvman.2023.119931 38154220 10.1016/j.jenvman.2023.119931

[19] Mendoza A, Aceña J, Pérez S et al (2015) Pharmaceuticals and iodinated contrast media in a hospital wastewater: a case study to analyse their presence and characterise their environmental risk and hazard. Environ Res 140:225–241. 10.1016/j.envres.2015.04.003 25880605 10.1016/j.envres.2015.04.003

[20] England A, Rawashdeh M, Moore N et al (2024) More sustainable use of iodinated contrast media—Why? Radiography (Lond) 30:74–80. 10.1016/j.radi.2024.06.023 38991461 10.1016/j.radi.2024.06.023

[21] National Inventory Report 2023 (2023) Greenhouse gas emissions and removals in Italy 1990–2021. ISPRA, Rome

[22] Seibert JA (2004) X-ray imaging physics for nuclear medicine technologists. Part 1: basic principles of x-ray production. J Nucl Med Technol 32:139–147 15347692

[23] Bushberg J, Seibert J, Leidholdt E, Boone J (2019) The essential physics of medical imaging, 4th edn. Wolters Kluwer, Philadelphia

[24] World Resources Institute, World Business Council for Sustainable Development (2015) GHG protocol scope 2 guidance. World Resources Institute (WRI)/World Business Council for Sustainable Development (WBCSD), Washington, DC

[25] International Energy Agency (2023) Electricity generation by source. International Energy Agency (IEA), Paris

[26] Nghiem DX, Yahyavi-Firouz-Abadi N, Hwang GL et al (2025) The iodine opportunity for sustainable radiology: quantifying supply-chain strategies to cut contrast’s carbon and costs. J Am Coll Radiol. 10.1016/j.jacr.2025.09.027 10.1016/j.jacr.2025.09.027 41046992

[27] Gerbens-Leenes W, Berger M, Allan J (2021) Water footprint and life cycle assessment: the complementary strengths of analyzing global freshwater appropriation and resulting local impacts. Water 13:803. 10.3390/w13060803

[28] International Organization for Standardization (2014) ISO 14046:2014: Environmental management — Water footprint —Principles, requirements and guidelines. Geneva: ISO; [accessed 2025 Sept 15]. https://www.iso.org/standard/43263.html

[29] Greffier J, Hamard A, Pereira F et al (2020) Image quality and dose reduction opportunity of deep learning image reconstruction algorithm for CT: a phantom study. Eur Radiol 30:3951–3959. 10.1007/s00330-020-06724-w 32100091 10.1007/s00330-020-06724-w

[30] Njølstad T, Jensen K, Dybwad A et al (2022) Low-contrast detectability and potential for radiation dose reduction using deep learning image reconstruction—a 20-reader study on a semi-anthropomorphic liver phantom. Eur J Radiol Open 9:100418. 10.1016/j.ejro.2022.100418 35391822 10.1016/j.ejro.2022.100418 PMC8980706

[31] Jensen CT, Liu X, Tamm EP et al (2020) Image quality assessment of abdominal CT by use of new deep learning image reconstruction: initial experience. AJR Am J Roentgenol 215:50–57. 10.2214/AJR.19.22332 32286872 10.2214/AJR.19.22332

[32] De Santis D, Polidori T, Tremamunno G et al (2023) Deep learning image reconstruction algorithm: impact on image quality in coronary computed tomography angiography. Radiol Med 128:434–444. 10.1007/s11547-023-01607-8 36847992 10.1007/s11547-023-01607-8 PMC10119038

[33] Schoen JH, Burdette JH, West TG et al (2024) Savings in CT net scan energy consumption: assessment using dose report metrics and comparison with savings in idle state energy consumption. AJR Am J Roentgenol 222:e2330189. 10.2214/AJR.23.30189 37937836 10.2214/AJR.23.30189

[34] Hehenkamp P, Obmann MM, Kamber S et al (2025) CT energy consumption savings from a rapid-reactivation power save mode for interexamination idle periods. AJR Am J Roentgenol. 10.2214/AJR.25.33951 10.2214/AJR.25.33951 41190835

[35] Carver DE, Pruthi S, Struk O et al (2025) Measuring the environmental impact of MRI and CT: a life cycle assessment. J Am Coll Radiol. 10.1016/j.jacr.2025.09.030 10.1016/j.jacr.2025.09.030 41052702

[36] Yoshida K, Nagayama Y, Funama Y et al (2024) Low tube voltage and deep-learning reconstruction for reducing radiation and contrast medium doses in thin-slice abdominal CT: a prospective clinical trial. Eur Radiol 34:7386–7396. 10.1007/s00330-024-10793-6 38753193 10.1007/s00330-024-10793-6

[37] Greenhalgh T, Wherton J, Papoutsi C et al (2017) Beyond adoption: a new framework for theorizing and evaluating nonadoption, abandonment, and challenges to the scale-up, spread, and sustainability of health and care technologies. J Med Internet Res 19:e367. 10.2196/jmir.8775 29092808 10.2196/jmir.8775 PMC5688245

[38] Bouza L, Bugeau A, Lannelongue L (2023) How to estimate carbon footprint when training deep learning models? A guide and review. Environ Res Commun 5:115014. 10.1088/2515-7620/acf81b 38022395 10.1088/2515-7620/acf81b PMC10661046

